# Dichloromethane Extracts of *Geranium Koreanum* Kom. Alleviates Esophagus Damage in Acute Reflux Esophagitis-Induced Rats by Anti-Inflammatory Activities

**DOI:** 10.3390/ijms19113622

**Published:** 2018-11-16

**Authors:** Hyeon Hwa Nam, Li Nan, Byung Kil Choo

**Affiliations:** Department of Crop Science & Biotechnology, Chonbuk National University, Jeonju 54896, Korea; hh_hh@jbnu.ac.kr (H.H.N); nanli5245988@gmail.com (L.N)

**Keywords:** acute reflux esophagitis, anti-inflammatory, claudin, gastroesophageal reflux disease, *Geranium koreanum*, NF-κB

## Abstract

Reflux esophagitis (RE) is a gastrointestinal disease caused by the reflux of gastric acid and stomach contents, and it leads to esophageal damage. Therefore, it is necessary to study the improvement of esophageal damage on a RE-induced model. The present study was accomplished to demonstrate the protective effects of a dichloromethane fraction of *Geranium koreanum* (DGK) plant on esophageal damage in an acute RE rat model. First, we examined the potential of anti-inflammatory effects of various fractions measured by cell cytotoxicity, morphological changes and nitric oxide (NO) production on lipopolysaccharide (LPS)-induced Raw 264.7 macrophage cells. Then, to evaluate the protective effects on RE, rats were partitioned into the following groups: normal control, RE-induced control and RE rats pre-treated with DGK 100 and 200 mg/kg body weight. The esophageal mucosal ulcer ratio was measured by the Image J program and histological changes were examined using a hematoxylin and eosin staining of the esophageal mucosa. The expression of pro-inflammatory proteins, cytokines and tight junction proteins involved in the esophageal mucosal damage were investigated using Western blotting and an enzyme-linked immunosorbent assay (ELISA) kit with esophagus tissue. DGK chemical profile and phenolic contents were analyzed by liquid chromatography-mass spectrometry (LC-MS/MS). The results showed that DGK exhibited anti-inflammatory effects against LPS-stimulated cells by significantly inhibiting NO production. Additionally, the results in vivo showed that improvement effects of DGK on esophageal mucosal damage. The expression of inflammatory proteins involved in nuclear factor κB (NF-κB) signaling pathways and tight junction protein (claudin-4 and -5) were significantly decreased in esophageal mucosa. We found the potential of DGK as source of replacement therapy products for inflammatory and RE disease.

## 1. Introduction

Gastroesophageal reflux disease (GERD) is usually induced by the reflux of gastric acid or stomach contents into the esophagus due to a defection of the lower esophageal sphincter and it related to unphysiological stress, excessive drinking, smoking, sleeping position, crapulence, and Westernization of the lifestyle and diet. Reflux esophagitis (RE) causes symptoms such as heartburn, nausea, chronic cough, pharyngeal pain and asthma and may lead to complication of Barrett esophagus, esophagus stenosis and esophageal cancer when the disease continues for a long time [1,2,3,4]. Proton pump inhibitors (PPIs) and histamine receptor antagonist forms of treatment have been widely used for rapid relief of symptoms and mucosal injury in patients with reflux disease, but these drugs cause many complications and are resistant to long-term use [5,6]. Therefore, recent research studies have focused on the development of safer alternatives that could be effective in the treatment of gastroesophageal disease. According to previous studies, herbal extracts act through various biological mechanism because they contain a wide range of bioactive compounds. It was reported that protective effects of herbal extracts on tissue damage are attributed to cellular defense mechanisms such as an inflammation and oxidative stress responses [7,8,9].

Inflammatory responses and tissue damage, the part of the physiological innate defense system that maintains immune homeostasis, are induced by inflammatory mediators [10,11,12]. Recently, reports about an inflammation such as mucosal damage in the esophagus due to reflux have been published. Souza et al. [13] demonstrated the pro-inflammatory chemokines secreted by reflux of gastric juice in the esophageal squamous epithelial cells. In addition, increased pro-inflammatory proteins such as tumor necrosis factor alpha (TNF-α), inducible nitric oxide synthase (iNOS) and cyclooxygenase-2 (COX-2) along with epithelium damage have been established in the esophageal tissue [14,15]. In addition, inflammatory changes associated with the pathogenesis of esophageal damage are induced by the release of gastric acid, which was found to be significantly increased in patients with RE [16,17].

Claudin, an integral membrane tight junction protein, functions as a barrier and maintains the polarity of epithelial cells. The changes of claudin protein expression have been demonstrated not only endothelium but numerous other cells and tissues, such as Schwann cells, olfactory epithelium, bowel mucosa, vascular endothelium, gastric cancer tissue and esophageal mucosa [18,19,20,21,22,23]. And, in the impaired mucosa, the loss of claudin proteins indicated by pro-inflammatory mediators induced cells or impaired mucosa.

*Geranium koreanum* Kom. is perennial plant from the Geraniaceae family and grows the Korean and northeast China. Geranium species have several phenolic compounds and commonly used as a conventional medicine to treat a various injury and symptoms such as diarrhea, itching, stomachic, intestinal inflammation and liver disorder, and antibacterial and hepatoprotective effects of *Geranium koreanum* extracts have been demonstrated [14,15,24,25]. Geraniin is the main polyphenolic compounds isolated from various Geranium species and is an important Chinese herbal medicine [26]. Previous studies have demonstrated that geraniin component has a wide range of pharmacological actions, including radioprotective, antioxidant, antitumor and inhibition effect on NF-κB activity [7,8,27,28]. Although the studies on for the beneficial anti-inflammatory effects of Geranium and Geraniin have been reported, none has sufficiently described the effect of *Geranium korenum* on esophageal damage. 

Therefore, the aim of the present study is to find the bioactive fraction in *Geranium koreanum* extracts and demonstrate the relive effect of fraction on esophageal tissue damage by recovering tight junction proteins and anti-inflammatory activities through the NF-κB signaling pathways. The anti-inflammatory effects of hexane (HEX), dichloromethane (DICHO), ethyl acetate (EA) and butanol (BUT) fractions were measured by morphological change, cell viability, NO and iNOS production in Raw 264.7 macrophage cells. In addition, we investigated the effect of DGK through gross esophageal damage ratio, histopathology analysis, and inflammatory protein expression analysis on the inflamed esophagus in experimental RE models.

## 2. Results

### 2.1. Cell Viability and Optical Morphological Transformation in Raw 264.7 Cells

**Figure 1:**
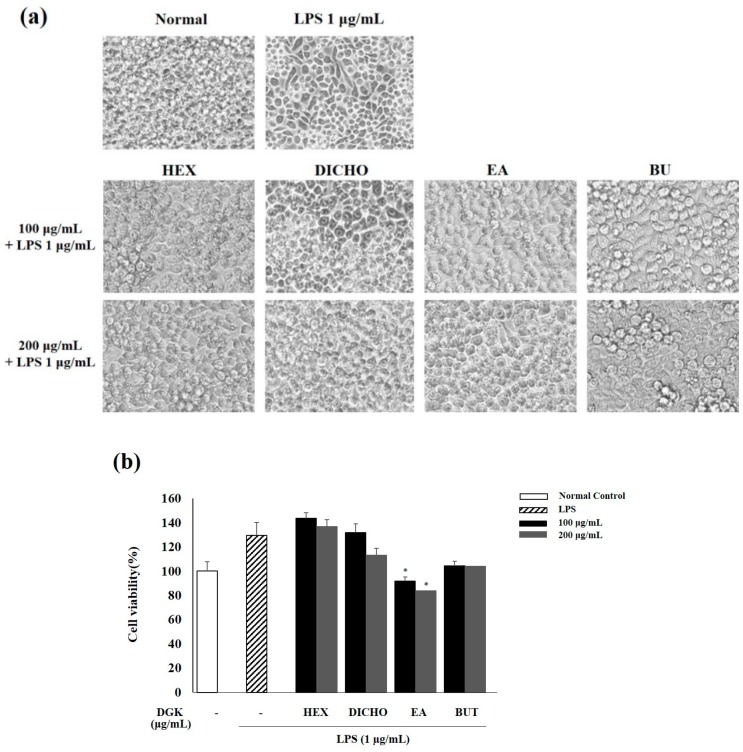
Optical morphological transformation (**a**) and cell viability (**b**) of on lipopolysaccharide (LPS) (1 µg/mL)-induced Raw 264.7 cells treated with the solvent fractions (HEX—hexane, DICHO—dichloromethane, EA—ethyl acetate and BUT—butanol) of *Geranium koreanum* (DGK) (100 and 200 µg/mL) for 24 h. The cell viability was determined using a cytotoxicity assay kit and showed no cytotoxicity. The morphology was visualized by a light microscope at 200× magnitude. Data are means ± standard deviation (SD); * *p* < 0.05 compared with compared with LPS control cells.

To identified the cytotoxicity of HEX, DICHO, EA and BUT fraction of *Geranium koreanum*, we analyzed the cell viability on Raw 264.7 macrophage cells treated with LPS (1 µg/mL) for 24 h using a cytotoxicity assay kit. The result showed that HEX, DICHO, EA and BUT fractions were not cytotoxicity against LPS-induced Raw 264.7 cells at concentrations of 100 and 200 µg/mL (Figure 1b). In the EA fraction, cell viability was slightly decreased compared to normal cells. Inflammation in LPS-induced Raw 264.7 macrophage cells lead to morphological changes, while untreated cells and samples kept their normal shape. We then investigated the potential effects of solvents fraction of *Geranium koreanum* on LPS (1 µg/mL)-induced transformation of Raw 264.7 cell morphology using a light microscope. LPS-induced inflammation significantly transformed the cell morphology, compared with that of normal cells cultured in a medium alone. Cells pre-treated with solvent fraction of *Geranium koreanum* (100 and 200 µg/mL), followed by LPS showed fewer transformed cells than the LPS treatment group (Figure 1a). In particular, the cells pre-treated with DICHO fraction demonstrated the most normal cell shape, compared with other fractions.

### 2.2. Effect of Geranium koreanum on Nitric Oxide (NO) Production and Inducible Nitric Oxide Synthase (iNOS) Expression in Lipopolysaccharide (LPS)-Induced in Raw 264.7 Cells

**Figure 2:**
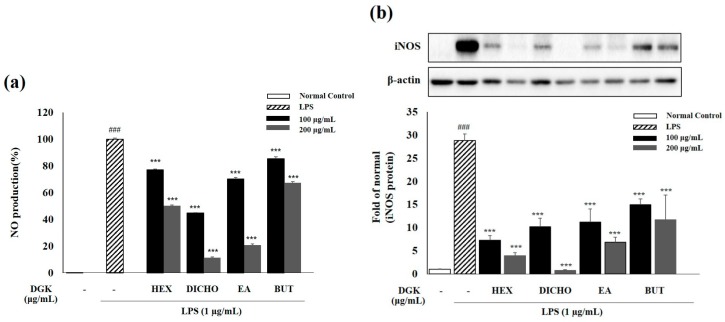
Inhibition of nitric oxide (NO) production (a) Inducible nitric oxide synthase (iNOS) protein expression in LPS (1 µg/mL)-induced Raw 264.7 cells pre-treated with 100 and 200 µg/mL solvent fractions (HEX—hexane; DICHO—dichloromethane; EA—ethyl acetate; BUT—butanol) of *Geranium koreanum* extracts for 24 h in 5% CO_2_ incubator. NO production was measured using the Griess assay and the expression of iNOS protein was analyzed using the Western blotting assay. Data are means ± standard deviation (SD); ^###^ *p* < 0.001 compared with normal control cells; *** *p* < 0.001 compared with LPS control cells.

We measured production of NO, a pro-inflammatory mediator, to determine whether the solvent fraction of *Geranium koreanum* had anti-inflammatory effects. NO plays a significant role in immune defenses and it is produced by iNOS during inflammatory responses in the cell or tissue [12]. The cells were pre-treated with the HEX, DICHO, EA and BUT fraction at concentrations of 100 and 200 µg/mL for 1 h, followed by LPS (1 µg/mL) treatment for 24 h. As shown in Figure 2, the NO production was hardly observed in the normal group, compared with in the LPS-treated group. In the cells pre-treated with the all solvent fractions of *Geranium koreanum* (100 and 200 µg/mL), NO production was significantly inhibited in cells treated with DICHO fraction of *Geranium koreanum* (100 and 200 µg/mL), compared with the LPS treated group; the effects were concentration-dependent. In particular, NO production decreased the most (56% and 89%) in cells treated with DICHO fraction (100 and 200 µg/mL) of *Geranium koreanum*. Also, expression of the pro-inflammatory mediator iNOS protein was significantly decreased in cells treated with DICHO fraction 200 µg/mL.

### 2.3. Improvement of Esophageal Mucosal Damage in Acute Reflux Esophagitis (RE) Rats 

**Figure 3:**
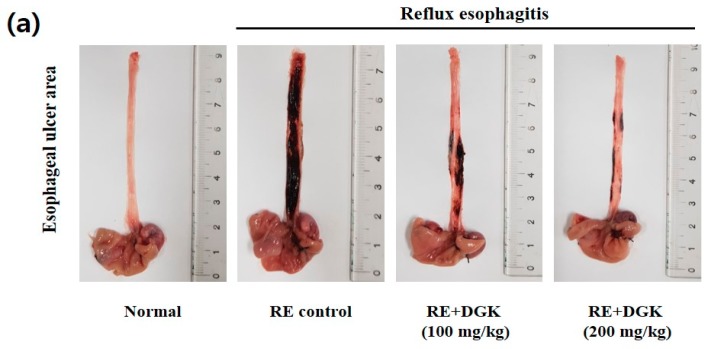

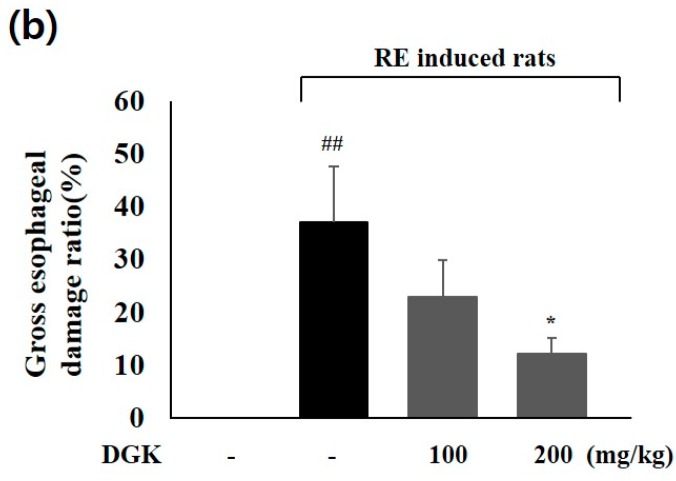
Image of gross esophageal erosions area (**a**) and esophageal mucosal ulcer ratio (**b**) of acute RE rats. Normal—untreated rats; reflux esophagitis (RE) Control—RE rats; RE + DGK100—DICHO fraction of *Geranium koreanum* 100 mg/kg body weight treated RE rats; RE + DGK 200—DICHO fraction of *Geranium koreanum* 200 mg/kg body weight treated RE rats. The incidence of erosions area was significantly decreased in rats treated with DGK. Data are means ± standard deviation (SD); ^##^ *p* < 0.01 and * *p* < 0.05 compared with normal control and RE control groups, respectively.

To evaluate the protective effects of DGK on RE rats, experimental rats received DGK (100 and 200 mg/kg body weight) orally 90 min before the surgery. Rats, except for those in the normal control group, were subjected to laparotomies to ligate the pylorus and the junction between the forestomach and the corpus [29]. Gastric acid reflux induced esophageal damage during the ligation. Esophageal tissue in the RE control group (untreated with sample) showed tissue damage (black and red color) and hemorrhage induced by inflammation, compared with those in the normal control group (Figure 3). The esophageal erosion area of rats in the RE + DGK200 (200 mg/kg) group significantly decreased (>68%) compared with that in the RE control group. Therefore, these results indicate that the DGK has protective effects against esophageal mucosal damage in acute RE rats.

### 2.4. Histopathology Analysis of Esophagus of RE Rats

**Figure 4:**
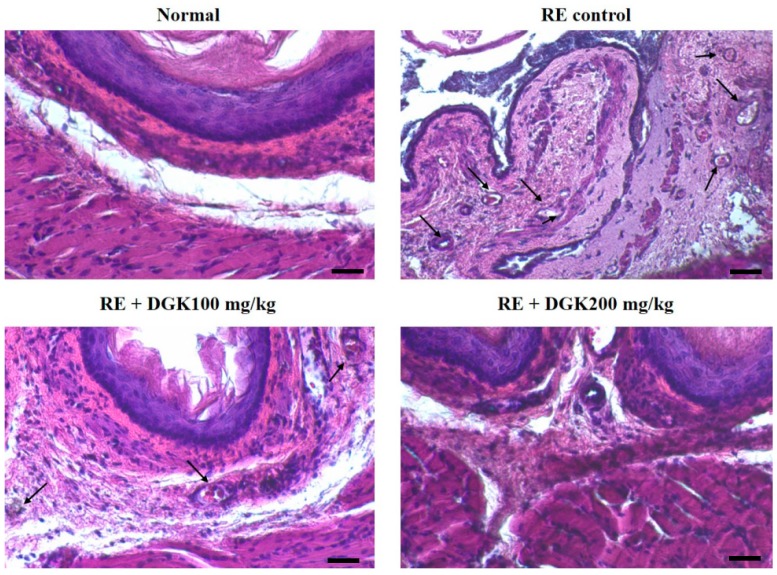
Hematoxylin and eosin staining of esophagus in RE rats. Normal control, reflux esophagitis (RE) control—RE + DGK 100 and RE + DGK 200 groups; Normal—untreated rats; RE Control—reflux esophagitis (RE) rats; RE + DGK100—dichloromethane fraction of *Geranium koreanum* 100 mg/kg body weight-treated RE rats; RE + DGK200—dichloromethane fraction *Geranium koreanum* 200 mg/kg body weight-treated RE rats (scale bar. 200 μM).

Esophageal tissue stained with hematoxylin and eosin showed no microscopic mucosal changes in normal control while more severe damages such as inflammatory cell infiltration, squamous cells, and a thin epithelial layer were observed in the mucosa and submucosa of the RE-induced rats. However, we observed that the esophagus damage of the RE rats was reduced to a similar condition to that of the normal control group by treatment with 200 mg/kg DGK. (Figure 4).

### 2.5. Inflammation-Related Protein Expression in Esophagus

**Figure 5:**
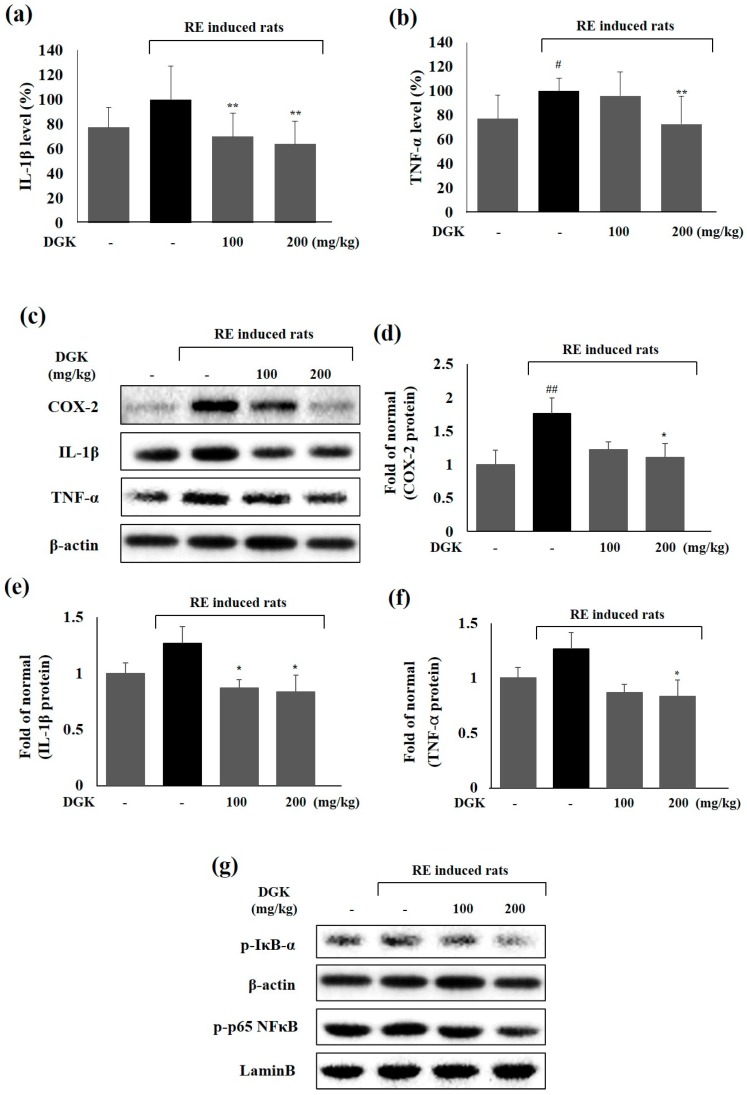

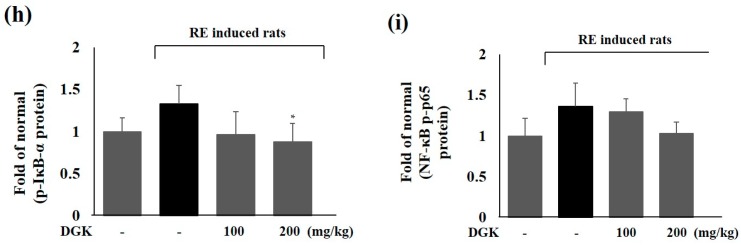
Inhibitory effects of DGK on inflammatory proteins and cytokines expression in esophagus. The expression levels of IL-1β (**a**) and TNF-α (**b**) in esophagus tissue were measured by enzyme-linked immunosorbent assay (ELISA). Expression of pro-inflammatory protein TNF-α, IL-1β, iNOS, COX-2, p-IκB-α, and p-p65 in esophagus tissue were measured by Western blotting (**c**–**i**). Rats were pre-treated with DGK 100 and 200 mg/kg body weight excluding normal and RE control for 90 min before surgery, which was performed under inhalation anesthesia. Then, 5 h later, the esophageal tissue was cut, and then, the was abdomen open and removed from each rat. Data are means ± standard deviation (SD). significance: ^##^ *p* < 0.01 and ^#^ *p* < 0.05 compared with normal control group; ** *p* < 0.01 and * *p* < 0.05 compared with RE control group.

Transcription factor NF-κB, which consists of p50 and p65, is the main regulator of the pro-inflammatory protein producing including iNOS, COX-2, cytokines TNF-α and interleukin-1β (IL-1β) during immune responses following its dissociation and phosphorylation by IκB-α. We identified pro-inflammatory mediators in the esophageal tissue by enzyme-linked immunosorbent assay (ELISA) kit and Western blotting to elucidate the expression of inflammatory proteins in inflamed tissue. In the ELISA assay, DGK showed inhibitory effect of the IL-1β and TNF-α protein increase on inflamed tissue (Figure 5a,b). Moreover, this inhibitory effect of DGK on the pro-inflammatory cytokines expression level were confirmed by western blot assay (Figure 5e,f). Phosphorylated-IκB-α (in the cytoplasm) and phosphorylated-p65 (in the nuclei) levels in the RE control group increased more than in the normal control group as a result of mucosal damage. Also, RE treatment increased pro-inflammatory protein expression, including COX-2, IL-1β, and TNF-α. DGK200 (200 mg/kg) improved esophageal inflammation by suppressing the expression of cytosolic p-IκB-α and nuclear p-p65 protein. In addition, DGK significantly reduced the increased expression of COX-2, TNF-α, and IL-1β protein, especially at 200 mg/kg (Figure 5c–i).

### 2.6. Claudin-4 and -5 Protein Expression in the Esophagus

**Figure 6:**
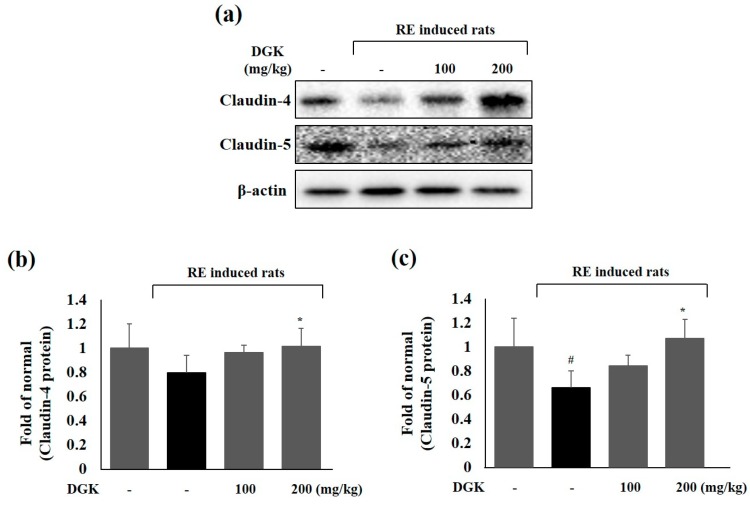
Expression of tight junction proteins claudin-4 (b) and claudin-5 (c) on esophagus in RE-induced rats (a). Rats were pre-treated with 100 and 200 mg/kg body weight DGK excluding normal and RE control for 90 min before surgery, which was performed under inhalation anesthesia. Then, 5 h later, the esophageal tissue was cut, and then, the was abdomen open and removed from each rat. Data are means ± standard deviation (SD). significance: ^#^ *p* < 0.05 and * *p* < 0.05 compared with normal control and RE control groups, respectively.

Claudin, one of the tight junction protein in the epithelium and endothelium, exert a critical barrier function of the tight junction. In particular, claudin-4 and claudin-5 play an important role because of their tightening or sealing functions in a tissue [21,30]. We observed the effects of DGK on tight junction proteins of the esophageal mucosa in an RE rat model (Figure 6a–c). In a previous study, as esophageal erosions decreased, the expression of claudin proteins increased in the esophageal mucosa of a rat model of with GERD [30]. However, the expression of claudin-4 and claudin-5 was increased in pre-treated DGK 200 mg/kg group in a concentration-dependent manner and was significantly higher than the expression in the RE control group.

### 2.7. Identifying Polyphenolic Compounds Using Liquid Chromatography-Mass Spectrometry (LC-MS/MS)

**Figure 7:**
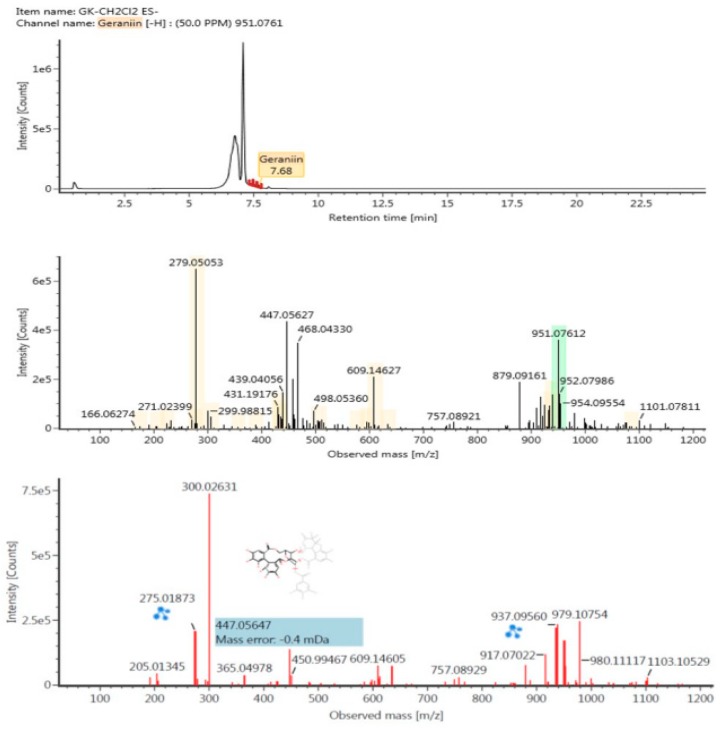
Identification of Geraniin in DGK by liquid chromatography-mass spectrometry (LC-MS/MS).

The polyphenolic profile of DGK was determined by liquid chromatography-mass spectrometry (LP-MS/MS). The comprehensive evaluation of DICHO fraction polyphenols allowed the identification of various compounds, the result shows the compounds separated by LC-MS/MS. The main compounds reported from *Geranium koreanum* was identified as Geraniin (retention time 7.68 min) (Figure 7). The detected compounds presented in Table 1. Based on the above data, the presence of the following compounds was identified in the DGK: ellagitannin (compounds 1, 6, 8, 10, 11), triterpenoid (compound 4), glycoside (compounds 2, 3, 9), and tannin (compound 7).

## 3. Discussion

In the present study, we found that DGK has anti-inflammatory effects via inhibition of NO production and iNOS protein expression in the LPS-induced macrophage. In addition, we found that the DGK is protected the esophagus in RE via suppression of pro-inflammatory protein expression and prevention of esophageal damage in an RE-induced experimental model.

Recently, studies on the use of herbal therapy in the management of reflux esophageal disease have been reported. Several studies have shown that traditional herbal was medications effectively ameliorated the histopathological signs of RE and decreased the esophageal injury index in the esophageal mucosa by regulating the expression of the inflammatory proteins and preventing the infiltration of inflammatory cells [1,3,7,18].

The geranium plant is well known as a traditional medicine to treat an inflammatory disease and it has various bioactive components. However, understanding of the varied bioactive effects such as the mechanism of the effect upon esophageal inflammation of *Geranium koreanum* (Geranium species) is still insufficient

LPS has been used to study induced infection, inflammation and biochemistry of inflammatory responses [30]. To demonstrate cytotoxicity and anti-inflammatory effects of different fractions, we cultured LPS-induced Raw 264.7 cells pre-treated with fraction samples were cultured for 24 h. All fractions of *Geranium koreanum* showed no significant cytotoxicity on Raw 264.7 cells (Figure 1b). Merely, in cells treated with EA fraction, cell viability was decreased slightly when compared with normal cells. Images of morphological changes of macrophages revealed that cell transformation in cells was increased by LPS stimulation; however, pretreatment with different fractions (especially, in the DICHO fraction) alleviated the morphological changes (Figure 1a). Base on this finding, we speculate that *Geranium koreanum* may be safely used as an herbal treatment for inflammatory disease. 

Moreover, to determine the potential bioactivity of a solvent fraction of *Geranium koreanum*, we evaluated the anti-inflammatory effects by measuring the changes in the NO production and iNOS expression. During inflammatory responses, the production of pro-inflammatory mediator NO is increased by the inducible isoforms of NO synthase (iNOS) proteins [31]. In LPS-induced macrophages cells treated with DICHO fractions (at concentrations of 200 µg/mL), different fractions displayed inhibitory effects of on NO production and iNOS expression in the following order DICHO > EA > HEX > BUT (Figure 2). The inhibition of NO production and iNOS protein expression by DICHO fractions (200 µg/mL) was indicated to be higher than that by the other fractions, and we identified that DICHO fraction showed the most potent anti-inflammatory activity among the different fractions.

Esophageal mucosal damage is caused by the reflux of gastric acid and stomach contents, which is related to the inflammation process and might lead to esophageal stenosis and cancer in the esophagus [3,32]. To evaluate the protective effects of DGK on RE rats, experimental rats were orally administrated with DICHO fraction (100 and 200 mg/kg body weight). In rats, except for those in the normal control group, reflux of stomach contents and gastric acid were induced into the esophagus. In this study, an esophageal tissue of the RE control group (untreated with sample) revealed tissue damage (black and red color) and hemorrhage, which were induced by inflammation responses, compared with the normal control group. The DGK100 (100 mg/kg) group showed a decreased incidence of damage in the esophagus. In the RE + DGK200 (200 mg/kg) group, the esophageal erosions area of rats significantly decreased by more than 68% compared with the RE control group (Figure 3a–b). The results of this study indicate that the DGK has attenuated esophageal mucosa damage in acute RE rats.

Tissue damage caused by exposure to acid and stomach contents increases infiltration of inflammatory cells in the epithelium of the esophagus [13,16]. As shown in Figure 4, various histological changes such as inflammatory cell infiltrations, edema, ulceration and exfoliation of epithelial cells were observed in esophageal tissues in RE-induced rats. Recently, several research works showed that similar pathological changes such as inflammatory infiltration, edema, hyperemia, and disruption and exfoliation of epithelial layers in RE-induced rats [7,8,13,33]. These changes showed that the relationship between the esophageal epithelium and the inflammatory response is a critical in the development of the esophageal damage ratio in RE. in the Thus, it can be suggested that inflammatory processes influence the underlying mechanisms that lead to of the symptoms and tissue damages in RE. In the group of administration of DGK to RE rats, DGK ameliorated inflammatory cell infiltration, hyperemia and disruption of epithelial layers in mucosa and submucosa. 

NF-κB, the main regulator of production of inflammatory proteins such as iNOS and COX-2, is activated by the cytokines tumor necrosis factor (TNF-α) and interleukin (IL)-1β during immune responses following its dissociation and phosphorylation by IκB-α. NF-κB has been reported to be involved in the expression of pro-inflammatory cytokines and proteins such as TNF-α, iNOS and COX-2 and is expressed in damaged esophagus mucosa in RE. Previous research has reported that following amelioration of esophageal damage by the extracts, the expression of inflammatory proteins was reduced in the esophagus [13]. In our study, we investigated to the expression of inflammatory biomarkers in damaged esophageal tissues to prove a relationship between esophageal mucosal damage and inflammatory responses and determine the effects of DGK. In the RE control group, the expression levels of both phosphorylated-IκB-α and phosphorylated-p65 protein were significantly increased after mucosal damage compared with the levels in a normal control group. The result of proteins level measurement showed that 100 and 200 mg/kg DGK improved the esophageal inflammation by inhibiting the expression of cytosolic p-IκB-α and nuclear p-p65 protein. In addition, DGK significantly affected the expression levels of iNOS, COX-2, TNF-α and IL-1β, especially at 200 mg/kg (Figure 5). Therefore, we demonstrated that the protective effects of DGK on mucosal damage in rats were related to regulating pro-inflammatory proteins in the esophagus. 

Claudin, a tight junction protein presents not only in the epithelium but also in the endothelium, is responsible for the critical barrier function of the tight junction. In particular, claudin-4 and claudin-5 have tightening or sealing functions in tissues [6,10]. In has been reported that pro-inflammatory cytokines/chemokines such as TNF-α, IL-6 and IL-10 induced loss of claudin-5 expression in cells. The expression of claudin-4 decreased in the RE control group compared with the drug treatment groups [10,34]. We observed the effects of DGK on tight junction proteins of the damaged esophageal mucosa in the RE rat model. We found that the expression of claudin-4 and claudin-5 were all increased concentration-dependently and was significantly higher in pre-treated DGK 200 mg/kg pretreated group than in the RE control group (Figure 6). Thus, we think that these regulatory effects abilities of DGK on inflammatory reaction and sealing functions in tissues could reduce esophageal damage induced by the reflux of gastric acid.

The polyphenolic profile of the DICHO fraction of *Geranium koreanum*, which has beneficial bioactivity, was detected by LC-MS/MS. Geraniin is the main phenolic compound of Geranium species, and it is reported to have bioactive effects such as anti-inflammatory effects via regulation of NF-κB and Nrf2 pathways in Raw 264.7 cells, cytoprotective effect via regulation of BACH-1 and HO-1 in HepG2 cells, and anticancer effects via inhibition of NF-κB activation [10,35,36,37,38,39,40,41]. Depending on the detector counts, the phenolic compounds of DGK are displayed in Table 1. Geraniin was present in the DICHO fraction at RT 7.68 min (Figure 7). It has been identified in *Geranium wilfordii* Maxim., *Geranium sibiricum* L., *Geranium robertianum* L., *Geranium thunbergii* [38,39,40,41].

The aim of our study was to demonstrate the ameliorating effects of bioactive DGK on esophageal damage caused by RE in vivo. We identified the potent inflammatory activity of DGK through changes in inflammatory biomarker expression in LPS-induced Raw 264.7 macrophages. In the RE rat model, DGK showed a protective effect on esophageal mucosal damage and decreased inflammatory cell infiltration and epithelial loss in the mucosa. In addition, we found that the defense mechanism underlying acid reflux-induced damage involved inflammatory processes, and DGK protected the esophageal mucosa by downregulating NF-κB pathway signaling. Moreover, the expression of tight junction proteins in the esophageal mucosa showed the tendency to increase following DGK treatment in RE rats. In the analysis by LC-MS/MS, the Geraniin component detected in the DGK exerts various bioactivities. In the study, we identified the protective effects of *Geranium koreanum* on RE and can suggest that it could be a safe alternative therapeutic source for inflammatory diseases.

## 4. Materials and Methods

### 4.1. Chemicals

LPS, dimethyl sulfoxide (DMSO), sulfanilamide, *N*-(1-naphthyl) ethylene-diamine dihydrochloride (NED), hematoxylin, eosin and protease inhibitor were purchased from Sigma-Aldrich (St. Louis, MO, USA), Dulbeco’s modified eagle’s media (DMEM), Fetal bovine serum (FBS) and penicillin/streptomycin were bought from Wel Gene (St. Namchen, Gyeongsan, Korea). Cyclohexane was purchased from Bychem (Yesan-gun, Chung nam, Korea). Methylene chloride was purchased from J.T. Baker (California, CA, USA), and n-butanol and ethyl acetate were bought from Sigma-Aldrich (St. Louis, MO, USA). The ELISA kit was purchased from R&D systems (Minneapolis, MN, USA). Primary antibodies lamin B was obtained from Cell Signaling (Danvers, MA, USA). iNOS, COX-2, β-actin, TNF-α, IL-1β, claudin-4, claudin-5, phospho-IκB-α and phospho-NF-κB were purchased from Santa Cruz (Dallas, TX, USA). The secondary antibodies goat anti-rabbit, goat anti-mouse and Western blotting luminal reagent were purchased from Santa Cruz. The bovine serum albumin (BSA) protein kit was bought from Bio-Rad Laboratories (Hercules, CA, USA). Geraniin was purchased from Chengdu Biopurify Phytochemicals Ltd. (Chengdu, China).

### 4.2. Plant Materials and Extraction

Fresh whole plants of *Geranium koreanum* were collected in Gangwon-do, Korea. Harvested *Geranium koreanum* was cleaned with water, dried at 50 °C for 24 h, and then a sample (500 g) was extracted three times with 70% ethanol for three times in 2 h. The extract was filtered and lyophilized (IlShin, Korea) and concentrated under reduced pressure to crude extracts using a rotary evaporator. The yield (*w*/*w* %) of ethanol extract was found to be 17.3%. This crude ethanol extract (80 g) was suspended in 1 L of distilled water and partitioned into various solvents (hexane, dichloromethane, ethyl acetate and butanol). The yields (*w*/*w* %) of the fraction were found to be 6.2% (4.96 g), 2.2% (1.76 g), 1.7% (1.36 g) and 8.4% (6.72 g) respectively. Hexane fraction was referred to as HEX, dichloromethane fraction as DICHO, ethyl acetate fraction as EA and butanol fraction as BUT. All solvents for the fraction were used purified. After preparing a fraction as a stock solution was stored at −20 °C and subsequently used as a sample for measurement.

### 4.3. Cell Culture with Geranium Koreanum Fractions

Raw 264.7 macrophage cell line was bought from the American Type Culture Collection (ATCC, Rockville, MD, USA). Raw 264.7 cells were maintained in DMEM supplemented with 10% FBS and 1% penicillin/streptomycin at 37 °C in a 5% CO_2_ incubator (SANTO, Sakata, Japan). The cells were pretreated with different concentrations (0, 100 and 200 µg/mL) of solvent fractions (HEX, DICHO, EA and BUT) for 1 h and then incubated with 1 µg/mL LPS for 24 h.

### 4.4. Cell Viability and Optical Morphological Transformation in Raw 264.7 Cells

To observed the impact of HEX, DICHO, EA and BUT fractions in cell viability and morphology transformation, cells were cultured in 96-well plates (1 × 10^6^ cells/well) and 6-well plates (5 × 10^5^ cells/well) treated with different concentrations (0, 100 and 200 µg/mL), followed by co-treatment with LPS (1 µg/mL) for 24 h at 37 °C in a 5% CO_2_ incubator. Cell viability was measured using a cytotoxicity assay kit, and the absorbance was measured at 540 nm using an ELISA plate reader (Multiscan Spectrum, Thermo Scientific, Vantaa, Finland). To observation of Raw 264.7 cells morphology transformation, the image of the cells was vidualized by an inverted microscope (ECLIPSE TS200, Nikon, Japan) at fixed 200× magnification.

### 4.5. Nitric Oxide (NO) Production in Raw 264.7 Cells

The production of NO in LPS-induced cells was measured as an indicator of NO production based on the Griess reaction. The cell culture plate conducted centrifugation process at 2500× *g*, 5 min. Then, 50 µL of the cell culture supernatant was mixed with 50 µL 1% sulfanilamide, and 0.1% NED incubated at 24 °C for 10 min. The absorbance at 540 nm was measured in an ELISA reader (multiscan spectrum). The amount of nitrite in the samples was determined using sodium nitrite serial dilution standard curve, and nitrite production was measured. 

### 4.6. Animal Management

The 7-week-old Sprague dawley (SD) rats were housed according to the animal welfare regulation of the Institutional Animal Care and Use Committee (IACUC; CBNU 2017-0078) at July 24, 2017, Chonbuk National University Laboratory Animal Center, South Korea. Rats were maintained in standard rat cages at temperature (23 ± 2 °C), humidity (35~60%) and photoperiod (12-h light/dark cycle), with food and water randomly provided.

### 4.7. Acute RE Induction

The 30 rats (7 weeks of age) used to establish the model were fasted for 18 h prior to the RE induction surgery; however, free access to distilled water was allowed. The rats were maintained in standard laboratory conditions (temperature 23 ± 2 °C, humidity 35~60% and photoperiod cycle 12 h light and 12 h dark). The rats were anesthetized using an inhalation anesthesia. A midline laparotomy was performed to expose the upper abdomen and a 3–0 silk thread was used to ligate the junction between the pylorus and the duodenum as well as the forestomach, (only sham control rats were not ligated). Following the surgery, 30 rats were randomly divided into four groups: (1) normal control rats, (2) RE control rats, (3) RE rats treated with DGK 100 mg/kg body weight, and (4) RE rats treated with DGK 200 mg/kg body weight (Table 2). The rats in the DGK groups were treated with 100 and 200 mg/kg DICHO fraction 90 min prior to the abdominal surgery. After 5 h, the samples were collected. Esophagus samples were collected and kept in −80 °C for Western blotting analysis and immediately fixed with 10% neutral-buffered formalin (NBF) for histological analysis.

### 4.8. Animal Management Esophageal Lesion Ratio

The rat esophagus was cut with scissors in a longitudinal direction from the gastroesophageal junction to the pharynx after sacrifice. The inner mucous was rinsed with saline solution, and the remaining tissue was placed on white paper. Then, the dissected esophagus was photographed using an optical digital camera (Nikon, Tokyo, Japan) and the images were analyzed using the Image J program. The gross mucosal damage ratio was calculated as follows: 

Gross mucosal damage ratio (%) = (width of the area with esophageal mucosal damage (mm^2^)/width of the total area of the esophagus (mm^2^)) × 100.

### 4.9. Histopathological Analysis of Esophageal Mucosa

Esophagus samples were excised for histopathological evaluation. Pieces of the esophagus of each rat was immediately fixed in 10% neutral-buffered formalin (NBF). After embedding in paraffin, 5-µm sections of the esophagus were stained with H-E and mounted on glass slides. Staining by hematoxylin and eosin was used to examine the histological architecture and apoptotic changes in the hepatocytes. Digital images of sample were obtained using a Leica DM2500 microscope (Leica Microsystems, Germany) at a fixed 200Xmagnification. The diameter of the portal vein was measured using image measurement software (i Solution DTM, v 22.1, Vancouver, Canada).

### 4.10. Preparation of Cytosol and Nuclear Fraction of Esophagus

Esophageal tissues were homogenized with ice-cold lysis buffer containing 10 mM 4-(2-Hydroxyethyl) piperazine-1-ehanesulfonic acid (HEPES) (pH 7.8), 2 mM magnesium chloride (MgCl_2_), 10 mM potassium chloride (KCl), and 0.1 mM ethylenediaminetetraacetic acid (EDTA), and then 1 mM dithiothreitol (DTT), 0.1 mM phenylmethylsulfonyl fluoride (PMSF) mixture solution and 10% NP-40 were added. After centrifugation (12,000× *g* for 2 min at 4 °C), the supernatant removed at each tube, and then the pellet was washed with distilled water. The nuclear proteins ware extracted from the esophagus, as described below. In brief, esophageal tissue was homogenized with ice-cold lysis buffer (pH 7.4) containing 50 mM HEPES (pH 7.8), 50 mM KCl, 300 mM NaCl, 1% glycerol, 0.1 EDTA, 1 mM PMSF, and protease inhibitor (PI) mixture solution. The samples were centrifuged at 12,000× *g* for 10 min at 4 °C. The protein concentration of each sample was measured using a Bio-Rad protein kit. 

### 4.11. Enzyme-Linked Immunosorbent Assay (ELISA) 

The levels of the pro-inflammatory cytokine IL-1β and TNF-α in the supernatants of esophagus tissue were evaluated by ELISA kits. The cytokines concentrations of the supernatant were determined in accordance with the manufacturer’s instructions and the standard curve.

### 4.12. Western Blot Analysis in Esophagus

The proteins extracted from the esophagus were separated by 10% and 12% sodium dodecyl sulfate-polyacrylamide gel electrophoresis (SDS-PAGE) and transferred from the gel to nitrocellulose membranes. The membrane was maintained with 5% skim milk in phosphate-buffered saline (PBS) solution 1% Tween 20 for 1 h at room temperature and incubated with the primary antibodies (1:1000) for overnight at 4 °C Then, the membrane incubated secondary antibodies (1:10000) at 2 h. Bands were detected using western blotting luminal reagent and images were obtained using a ChemiDocTM MP imaging system (Bio-Rad).

### 4.13. LC-MS/MS analysis

The phenolic compounds of DICHO isolated from *Geranium koreanum* analysis was performed using an ultra-performance liquid chromatography (UPLC) system (Waters, Milford, CT, USA). The conditions of LC-MS/MS analysis are provided Table 3.

### 4.14. Statistical Analysis

All results are expressed as mean ± SD of at least three independent experiments. Statistical analysis was performed using the statistical package of the social sciences (SPSS) software (SPSS Inc., Chicago, IL, USA) using analysis of variance (ANOVA), followed by Tukey’s multiple comparison tests. Differences were considered significant at *p* < 0.05

## Figures and Tables

**Table 1:** Identification of phytochemical compounds in DGK by LC-MS/MS.

No.	Component Name	Neutral Nass (Da)	Observed Neutral Mass (Da)	Detector Counts	Adducts	Group
1	6′-*O*-Galloyl-homoarbutin	438.1192	438.1162	149,619	−H	Ellagitannin
2	Chlorogenin	432.324	432.3236	470,753	−H	Glycoside
3	Markogenin	432.324	432.3236	470,753	−H	Glycoside
4	23-Acetate alisol E	532.3764	532.3758	35,139	+HCOO	Triterpenoid
5	9,16-Dioxyhydroxy-10,12,14-triene-18 carbonic acid	310.2144	310.214	33,679	−H	Terpene
6	Castalagin	934.0712	934.0725	11,056	−H	Ellagitannin
7	Euphormisin M2	924.0869	924.0882	19,280	+HCOO, −H	tannin
8	Geraniin	952.0818	952.0834	18,307	−H	Ellagitannin
9	Koryoginsenoside R1	868.5184	868.5195	14,293	−H	Glycoside
10	Koryoginsenoside R1	868.5184	868.5194	10,068	−H	Glycoside
11	Sanguiin H-4	634.0806	634.0809	12,355	−H	Ellagitannin

**Table 2:** The body weight of the 30-rat model.

	Group	Body Weight (g)
1	Normal (*n* = 6)	225.0 ± 4.328
2	Control (*n* = 8)	226.6 ± 2.171
3	DICHO 100 mg/kg (*n* = 8)	224.6 ± 2.104
4	DICHO 200 mg/kg (*n* = 8)	220.7 ± 2.179

Mean body weight of rats (means ± standard deviation (SD)).

**Table 3:** The condition of LC-MS/MS analysis.

Parameter		Condition		
Ultra-performance liquid chromatography (UPLC)		ACQUITY UPLC HSS T3		
Column		100 mm × 2.1 mm, 1.8 µm, Waters		
Column temperature		40 °C		
Flow rate		0.5 mL/min		
mobile phase		A (water + 0.1% formic acid)		
		B (acetonitrile + 0.1% formic acid)		
		Time	A (%)	B (%)
		0	97	3
		5	97	3
		16	0	100
		17	0	100
		19	97	3
		25	97	3
injection volume		5 µL		
metabolite eluted		SYNAPT G2 Si HDMS QTOF (Waters), positive and negative		
positive	capillary voltage (kV)	2		
	cone voltage (V)	40		
negative	capillary voltage (kV)	1		
	cone voltage (V)	40		
scan range (Da)		50 to 1200		
scan time (s)		0.2		
